# The cholinesterase and C-reactive protein score is a potential predictor of pseudoaneurysm formation after pancreaticoduodenectomy in patients with soft pancreas

**DOI:** 10.1186/s12893-023-02211-3

**Published:** 2023-11-14

**Authors:** Yoshifumi Morita, Takanori Sakaguchi, Akio Matsumoto, Shinya Ida, Ryuta Muraki, Ryo Kitajima, Satoru Furuhashi, Makoto Takeda, Hirotoshi Kikuchi, Yoshihiro Hiramatsu, Hiroya Takeuchi

**Affiliations:** 1 Department of Surgery, Hamamatsu University School of Medicine, 1-20-1 Handayama, Higashi-ku, Hamamatsu, 431-3192 Japan; 2 Division of Surgical care, Morimachi, Hamamatsu University School of Medicine, Hamamatsu, Japan; 3 Department of Surgery, Iwata City Hospital, Iwata, Japan; 4 Department of Perioperative Functioning Care & Support, Hamamatsu University School of Medicine, Shizuoka, Japan

**Keywords:** Pseudoaneurysm, Pancreaticoduodenectomy, C-reactive protein, Cholinesterase

## Abstract

**Background:**

Pseudoaneurysm (PA) rupture after pancreaticoduodenectomy (PD) is a life-threatening complication. Most PA cases originate from postoperative pancreatic fistulas (POPFs). Although several risk factors for POPF have been identified, specific risk factors for PA formation remain unclear. Therefore, we retrospectively analyzed PD cases with soft pancreas and proposed a novel strategy for early detection of PA formation.

**Methods:**

Overall, 120 patients underwent PD between 2010 and 2020 at our institution; of these, 65 patients with soft pancreas were enrolled. We evaluated the clinicopathological factors influencing PA formation and developed a risk score to predict PA formation.

**Results:**

In total, 11 of the 65 patients developed PAs (PA formation group: PAG), and 8 of these 11 PAs ruptured. The median time to PA formation was 15 days, with a minimum of 5 days. The PAG was significantly older than the non-PA formation group, were predominantly men, and had comorbid diabetes mellitus. Pre- and intra-operative findings were similar between the two groups. Importantly, no significant differences were found in postoperative drain amylase levels and total drain amylase content. Cholinesterase and C-reactive protein (CRP) levels on postoperative day (POD) 3 were significantly different between the two groups. Multivariate analysis showed that cholinesterase ≤ 112 U/L and CRP ≥ 16.0 mg/dl on POD 3 were independent predictors of PA formation.

**Conclusions:**

Decreased cholinesterase and elevated CRP on POD 3 (Cho-C score) are useful predictors of PA formation in cases with soft pancreas. In such cases, periodic computed tomography evaluations and strict drain management are necessary to prevent life-threatening hemorrhage.

**Supplementary Information:**

The online version contains supplementary material available at 10.1186/s12893-023-02211-3.

## Introduction

Pancreaticoduodenectomy (PD) is a highly invasive procedure. According to the national clinical database in Japan, the overall complication and in-hospital mortality rates were 40.0% and 2.8%, respectively [1]. Similarly, according to the database from American College of Surgeons-National Surgery Quality Improvement Program (ACS-NSQIP), the overall complication and mortality rates were 31.8% and 2.5%, respectively [2]. Recent studies have focused on failure to rescue (FTR) rather than complication rates. FTR is perioperative mortality due to serious complications and is thought to be an indicator of complication management and hospital performance [3]. Multi-institutional study from 23 international expert centers in pancreas surgery reported the benchmark of severe complications and FTR were 30% and 9%, respectively [4].

Postoperative pancreatic fistula (POPF) is a major complication after PD. It is defined from the drain amylase level on or after postoperative day (POD) 3 according to the definition of the International Study Group of Pancreatic Surgery (ISGPS) [5]. Although gender, age, BMI, and intraoperative blood loss have been listed as risk factors for POPF, the risk of POPF primarily depends on the pancreatic parenchyma and pancreatic duct diameter [6, 7]. Clinically relevant POPF rarely occurs in inflamed, obstructed pancreas, which is the so-called hard pancreas. In contrast, POPF occurs with a certain frequency in patients with soft pancreas. POPF can cause severe complications such as intra-abdominal hemorrhage and sepsis [8].

Post-pancreatectomy hemorrhage (PPH) is a life-threatening complication, and late onset PPH is mainly caused by pseudoaneurysm (PA) rupture [9]. Mortality rates for PPH patients have been reported to range from 16 to 50% [10–12]. Since most PA cases originate from severe POPF, detection at the stage of unruptured PA formation would be useful. Several risk factors for POPF have been identified, but the specific risk factors for PA formation remain unclear.

This study hypothesized that peri-operative clinicopathologic factors, which include nutritional indicators affecting wound healing, contain predictors of PA formation. Therefore, we retrospectively analyzed PD cases with soft pancreas, aiming to develop a novel strategy for early detection of PA formation.

## Methods

### Patients

**Fig. 1:**
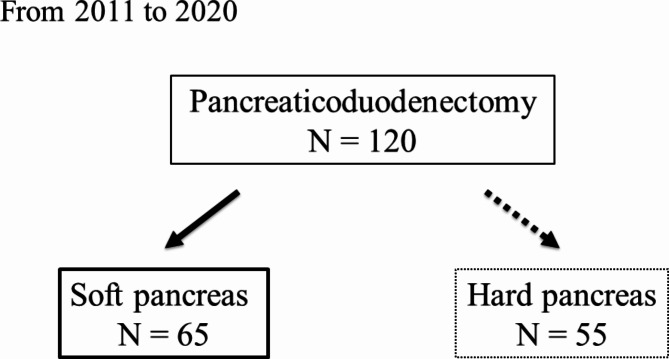
Study population

Between 2010 and 2020, 120 patients underwent PD at the Hamamatsu University School of Medicine. Of these, 55 patients with hard pancreas were excluded from the analysis. A pancreas with a main pancreatic duct diameter of ≤ 3 mm was defined as a soft pancreas. Furthermore, even if the main pancreatic duct was dilated, the pancreas was judged to be soft by the surgeons if no inflammation was found in the pancreatic parenchyma. Soft pancreas included friable and brittle tissue, as defined by ISPGS [13]. Therefore, a total of 65 patients were enrolled in this study (Fig. 1).

### Surgical procedure

Overall, 14 surgeons performed PD in this study, and the number of cases per surgeon ranged from 1 to 44. All surgeons had experience in general and gastrointestinal surgery of > 5 years. Expert surgeons certified by the Japanese Society of Hepato-Biliary Pancreatic Surgery participated in all cases, either as surgeons or first assistants. Lymphadenectomy for malignancy included anterior and posterior pancreaticoduodenal, pyloric region, hepatoduodenal ligament, common hepatic artery, and superior and inferior pancreatic head lymph nodes. Nerve plexus dissection around the superior mesenteric artery was performed according to the location and extent of the tumor. Simultaneous superior mesenteric vein (SMV) and portal vein (PV) resection was performed when the involved SMV and PV could be safely reconstructed by direct suture or venous graft. Gastrointestinal reconstruction was performed with the modified Child method. Pancreatic reconstruction was mainly performed with pancreaticojejunostomy, duct-to-mucosa anastomosis. Fibrin-glue was routinely used at the pancreaticojejunostomy site. Choledochojejunostomy was performed 5 to 10 cm on the anal side of the pancreaticojejunostomy. In most cases, external drainage tubes were placed at the pancreaticojejunostomy and choledocojejunostomy sites. Gastrojejunostomy or duodenojejunostomy were performed via the antecolic route. Prophylactic intra-abdominal drains were placed around the pancreatic and biliary anastomoses. Closed and continuous suction drains were routinely placed in the Winslow foramen and around the pancreaticojejunostomy site. The round ligament of the liver was wrapped from the common hepatic artery to the proper hepatic artery to cover the stump of the gastroduodenal artery. From 2018, peritoneal lavage was performed with 10 L of saline. Tube ileostomy was not routinely performed, except in high-risk patients.

### Peri-operative management

Routine blood tests were performed on PODs 1, 2, 3, 5 and 7. Drain amylase levels were routinely measured on PODs 1, 2, and 3. Blood test and measurement of drain amylase levels were subsequently performed according to the postoperative course. If pre-operative biliary drainage was performed, prophylactic antibiotics were selected according to the bile culture results. Prophylactic antibiotics were used until POD 2. In contrast, if an intra-abdominal infection was suspected, broad-spectrum antibiotics were administered until culture results were obtained. A somatostatin analogue was used immediately after surgery in cases with soft pancreas and when postoperative drain amylase levels were high. Previously, computed tomography (CT) examination was performed in cases with high fever, elevated C-reactive protein (CRP) concentration, or contaminated or bloody drainage fluid. From 2018, CT examination was routinely performed on PODs 4 or 5. Surgically placed abdominal drains were aggressively exchanged if drain amylase levels were high or the drainage fluid was contaminated. Exchanged drains were washed with saline during the morning and evening rounds or with continuous saline irrigation and suction.

### Diagnosis and treatment of pseudoaneurysms

**Fig. 2:**
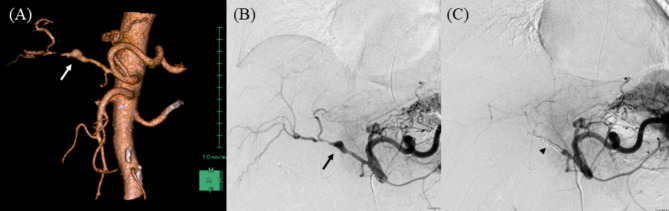
Representative images of a pseudoaneurysm after pancreaticoduodenectomy (Unruptured) **(a)** Three-dimensional reconstruction image of contrast-enhanced computed tomography. The white arrow indicates a pseudoaneurysm and vascular irregularity **(b)** Emergency angiographic image from the celiac artery. The black arrow shows a pseudoaneurysm and post-dilation stenosis **(c)** Angiographic image after coil embolization (black arrowhead)

An increased arterial diameter, with active bleeding on enhanced CT scan, was defined as PA formation and rupture. Obvious arterial irregularity, judged by the radiologists to require urgent treatment, were defined as PA formation (Fig. 2). Coil embolization was mainly undertaken in cases with active bleeding and unstable vital signs. However, stent graft placement was considered if the vital signs were stable and bleeding was minimal or absent.

### Classification of postoperative complications

The definition and grading of ISGPS was used to classify POPF, PPH, and chyle leak [5, 9, 14]. The Clavien–Dindo surgical complication classification was used to classify postoperative complications [15].

### Fistula risk score

According to the previous report, the fistula risk score was calculated using parameters such as pancreatic texture, pathology, pancreatic duct diameter, and intraoperative blood loss [6].

### Pre-operative nutritional assessment

The Geriatric nutritional risk index (GNRI) was defined according to an earlier report [16] as follows: [1.489 * albumin (g/dL)] + [41.7 * (weight/ideal weight).

The modified Glasgow prognostic score (mGPS) was defined according to an earlier report [17] as follows: patients with an elevated CRP concentration (1 mg/dL) and a decreased albumin concentration (3.5 g/dL) were assigned a score of 2; those with an elevated CRP concentration (1 mg/dL) were assigned a score of 1; and those with a CRP concentration of < 1 mg/dL and any albumin concentration were assigned a score of 0.

The prognostic nutritional index (PNI) was defined as follows : [10 * albumin (g/dL)] + [0.005 * total lymphocyte count (/mm^3^)] [18].

### Statistical analysis

All continuous data were expressed as medians (min-max). The Mann-Whitney U test was used to compare continuous variables. Pearson’s chi-squared test or Fisher’s exact test were used to compare categorical variables. The optimal cutoff values to discriminate between the PA formation group (PAG) and the non-PA formation group (NPAG) were determined using receiver operating characteristic (ROC) curve analysis. The area under the curve (AUC) was calculated to validate the discrimination abilities of the candidate. Multivariate logistic regression analysis was performed to identify independent factors from multiple candidates. Statistical significance was considered at p < 0.05, and SPSS version 27 (IBM Corp., Armonk, NY, USA) was used for all statistical analyses.

## Results

### Characteristics of the study cohort

From 2011 to 2020, we had 120 PD cases, including 65 cases with soft pancreas. Table 1 presents the patients’ basic clinicopathological characteristics. The median age was 70, and approximately two-thirds were men. The main primary diseases were non-pancreatic malignancy, including 25 biliary tract cancers, 6 neuroendocrine tumors, and 4 duodenal cancers because of the nature of the study cohort. Half of the patients underwent pre-operative biliary drainage. Since we adopted aggressive drain management, particularly in higher drain amylase cases, the number of clinically relevant POPFs was 43 of the 65 cases (66.1%). The main reason for POPF grade B was persistent drainage for ˃3 weeks. We experienced 11 cases of PA formation, including 8 ruptured and 3 unruptured cases. Ruptured cases were noticed by bleeding from drains, and unruptured cases were incidentally identified by postoperative CT. The sites of PA formation were the common hepatic artery (CHA), the stump of the gastroduodenal artery, the CHA to the proper hepatic artery, and the PHA in 4, 3, 2. and 2 cases, respectively. The median time of PA formation was 15 days after PD. Importantly, the earliest onset of PA rupture was only 5 days after PD. Conversely, four cases of pseudoaneurysms that developed more than one month after surgery were also included. PPH grades B and C, according to the ISGPF definition, occurred in 1 and 7 cases, respectively. No early onset PPH was observed in this study cohort. However, 1 of the 7 grade C cases (14%) died of hemorrhagic shock on POD 7.

**Table 1:** Study cohort characteristics

	Soft pancreas N = 65
Age (min-max) (years)	70 (26–84)
Sex (Male : Female)	41 : 24
ASA score (1 : 2 : 3)	7 : 49 : 9
Pre-operative BMI (kg/m^2^) (min-max)	22.3 (16.3–30.8)
Primary disease (Pancreatic cancer : non-pancreatic malignancy^†^ : others)	21 : 35 : 9
Neoadjuvant chemotherapy (Y : N)	2 : 63
Pre-operative biliary drainage (Y : N)	33 : 32
POPF grade (none : BL: B : C)	9 : 13 : 38 : 5
PA formation (Y : N)	11 : 54
Site of PA formation (CHA : GDA stump : CHA ~ PHA : PHA)	4 : 3 : 2 : 2
Time of PA formation post-operation (days)	15 (5–70)
PPH (Y : N)	8 : 57
PPH grade (A : B : C)	0 : 1 : 7*

ASA: American Society of Anesthesiologists, BMI: Body mass index, CHA: Common hepatic artery, GDA: Gastroduodenal artery, PHA: Proper hepatic artery, PA: Pseudoaneurysm, POPF: Postoperative pancreatic fistula, PPH: Postpancreatectomy hemorrhage

^†^ Including 25 biliary tract cancers, 6 neuroendocrine tumors, and 4 duodenal cancers

* Including one case of in-hospital death

### Comparison of peri-operative clinicopathological characteristics

Table 2 presents the clinicopathological characteristics stratified by PA formation. The median age in the PAG was significantly higher than that in the NPAG. In this study, PA formation only occurred in men. Although the American Society of Anesthesiologists scores were similar between the two groups, most of the PAG had some systemic comorbidity. Diabetes mellitus was more frequently observed in the PAG. Cardiovascular complications or hypertension were tended to be more frequent in the NPAG. Primary disease, neoadjuvant chemotherapy, pre-operative biliary drainage, and pre-operative cholangitis were not significantly different between the two groups. Intra-operative findings, including operation time, blood loss, pancreatic duct size, concomitant resection of other organs, lymph node dissection, and venous reconstruction rates, were not significantly different between the two groups. The fistula risk score was not different between the two groups. Furthermore, clinically relevant POPF occurred in all cases of the PAG. Postoperative bile leak and intraabdominal infection were not significantly different between the two groups. The above-mentioned results were similar when the subject was limited to patients with POPF (Supplementary Table 1).

**Table 2:** Peri-operative clinicopathological characteristics

	PAG N = 11	NPAG N = 54	*p-value*
Age (min-max) (years)	74 (60–82)	68 (26–84)	**0.048**
Age > 70 (Y : N)	9 : 2	25 : 29	**0.047**
Sex (Male : Female)	11 : 0	30 : 24	**0.005**
Comorbidities (Y : N)	10 : 1	32 : 22	0.080
Diabetes mellitus (Y : N)	5 : 6	7 : 47	**0.011**
Cardiovascular disease (Y : N)	1 : 10	25 : 29	**0.039**
Hypertension (Y : N)	1 : 10	21 : 33	0.082
ASA score (1 : 2 : 3)	0 : 9 : 2	7 : 40 : 7	0.432
Pre-operative BMI (kg/m^2^) (min-max)	22.4 (19.3–26.8)	21.6 (16.3–30.8)	0.780
Primary disease (Pancreatic cancer : non-pancreatic malignancy^†^ : others)	3 : 6 : 2	18 : 29 : 7	0.867
Neoadjuvant chemotherapy (Y : N)	0 : 11	2 : 52	1.000
Pre-operative biliary drainage (Y : N)	6 : 5	27 : 27	0.783
Pre-operative cholangitis (Y : N)	3 : 8	6 : 48	0.157
Operation time (min) (min-max)	417 (314–652)	420 (273–754)	0.354
Blood loss (ml) (min-max)	705 (194–1520)	534 (70–3290)	0.278
Pancreatic duct size (min-max)	3 (2–4)	3 (2–12)	0.762
Fistula risk score (min-max)	6 (4–9)	6 (2–9)	0.319
Bile duct size (mm) (min-max)	7.5 (4–13)	8.0 (3–24)	0.751
Concomitant other organ resection (Y : N)	0 : 11	5 : 49	0.579
SMA nerve plexus dissection (Y : N)	1 : 10	13 : 41	0.492
Lymph node dissection (D2 : D1 or D0)	10 : 1	47 : 7	1.0000
Venous reconstruction (Y : N)	1 : 10	3 : 51	0.533
Tube ileostomy (Y : N)	0 : 11	9 : 45	0.337
Blood transfusion (Y : N)	0 : 11	6 : 48	0.579
Clinically relevant POPF (Y : N)	11 : 0	32 : 22	**0.011**
Chyle leak (Y : N)	0 : 11	0 : 54	1.000
Bile leak (Y : N)	0 : 11	1 : 53	1.000
Intraabdominal infection (Y : N)	6 : 5	25 : 29	0.618
Complication (C.D. grade ≥ III) (Y : N)	11 : 0	35 : 19	**0.025**
Mortality (Y : N)	1 : 10	0 : 54	0.169

ASA: American Society of Anesthesiologists, BMI: body mass index, C.D.: Clavien-Dindo, NPAG: non-PA formation group, PAG: PA formation group, POPF: postoperative pancreatic fistula, SMA: superior mesenteric artery,

^†^ Including 25 biliary tract cancers, 6 neuroendocrine tumors, and 4 duodenal cancers

### Pre-operative blood test results

We compared the pre-operative blood test results to identify predictive markers of PA formation (Table 3). However, the two groups had similar blood counts, blood chemistry, and clotting functions. Furthermore, various nutritional index, including GNRI, mGPS, neutrophil to lymphocyte ratio, lymphocyte to monocyte ratio, platelet to lymphocyte ration, and PNI were not significantly different between the two groups.

**Table 3:** Pre-operative blood test results

	PAG N = 11	NPAG N = 54	*p-value*
BUN (mg/dl)	13.9 (7.2–20.1)	14.1 (6.5–52.3)	0.965
Cre (mg/dl)	0.75 (0.51–1.20)	0.75 (0.36–8.18)	0.655
T. bil (mg/dl)	0.7 (0.3–5.3)	0.8 (0.2–6.8)	0.785
T. cholesterol (mg/dl)	159 (134–247)	183 (109–346)	0.469
AST (U/L)	25 (16–156)	30 (5–140)	0.746
ALT (U/L)	18 (9–239)	31 (4–382)	0.441
Cholinesterase (U/L)	240 (169–298)	269 (121–708)	0.137
AMY (U/L)	88 (58–142)	81 (28–210)	0.431
Alb (g/dl)	3.9 (3.2–4.2)	4.1 (2.8–4.9)	0.157
GNRI	99.1 (84.6–111.8)	103.3 (77.0–126.5)	0.336
HbA1c (%)	6.8 (5.6–7.8)	5.8 (4.5–8.5)	**0.002**
CRP (mg/dl)	0.14 (0.03–6.61)	0.14 (0.01–3.33)	0.993
mGPS (0 : 1 : 2)	10 : 1 : 0	47 : 6 : 0	1.000
WBC (/µl)	5620 (3000–23,700)	5255 (2610–9080)	0.637
Neutrophil (/µl)	3340 (1620–7949)	3274 (1461–5984)	0.956
Lymphocyte (/µl)	1300 (551–2418)	1325 (600–2802)	0.631
Monocyte (/µl)	334 (201–499)	340 (142–692)	0.739
Neutrophil to Lymphocyte ratio (NLR)	2.5 (1.0–7.0)	2.4 (0.9–7.6)	0.698
Lymphocyte to monocyte ratio (LMR)	3.6 (1.8–7.1)	3.6 (1.7–8.1)	0.810
Hb (g/dl)	13.9 (9.7–14.9)	13.4 (7.8–16.9)	0.393
PLT ( ×10^4^/µl)	25.1 (10.3–43.5)	21.2 (8–46.4)	0.506
Platelet to Lymphocyte ratio (PLR)	220.1 (56.7–361.1)	151.2 (46.2–705.2)	0.222
Prognostic nutritional index (PNI)	44.6 (39.8–51.1)	47.3 (31.7–59.3)	0.222
PT (%)	114.0 (77.0–132.0)	102.0 (70.0–140.0)	0.624
APTT (%)	92.0 (70.0–126.0)	91.0 (46.0–140.0)	0.755

CRP: C-reactive protein, GNRI: Geriatric nutritional risk index, mGPS: modified Glasgow prognostic score, PA: pseudoaneurysm, PAG: PA formation group, NPAG: non-PA formation group

### Comparison of postoperative blood and drain test results

**Fig. 3:**
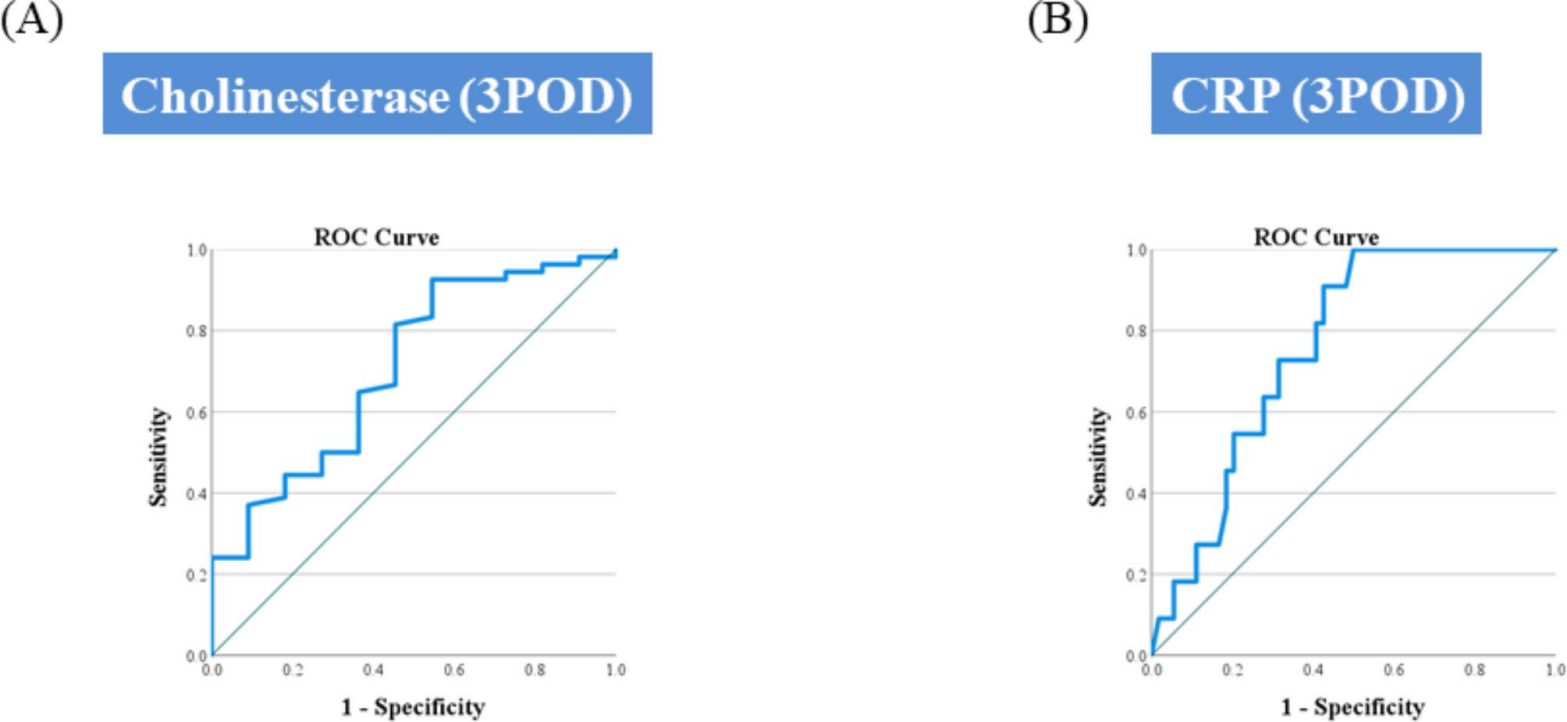
ROC curve discriminating pseudoaneurysm formation Cholinesterase level **(a)** and CRP level **(b)** on POD 3. The X- and Y-axes show the sensitivity and specificity of pseudoaneurysm formation, respectively. ROC: receiver operating characteristic; CRP: C-reactive protein; POD: postoperative day

We performed daily blood tests and examined drain amylase levels from PODs 1 to 3. On POD 1, only the CRP level was significantly higher in the PAG (Table 4). On POD 2, the CRP maintained its higher level in the PAG. Furthermore, cholinesterase levels tended to be lower in the PAG (Table 5). Finally, CRP and cholinesterase levels on POD 3 were significantly higher and lower in the PAG, respectively. (Table 6). Importantly, drain amylase levels from PODs 1 to 3 were not significantly different between the two groups. Additionally, the total drain amylase content, the sum of each drain amylase level multiplied by the amount of drain fluid, was also not significantly different between the two groups. These results were similar when the subject was limited to patients with POPF (Supplementary Tables 2, 3). The ROC curve showed a cutoff level of cholinesterase, on POD 3, of 112 U/l, with a sensitivity, specificity, and AUC of 81.5%, 54.5%, and 0.708 (95% confidence interval [CI]:0.539–0.877), respectively (Fig. 3a). The ROC curve showed a cutoff level of CRP on POD 3, of 16.0 mg/dl, with a sensitivity, specificity, and AUC of 90.9% 55.6%, and 0.758 (95% CI:0.635–0.882), respectively (Fig. 3b).

**Table 4:** Results of blood and drain tests on postoperative day 1

	PAG N = 11	NPAG N = 54	*p-value*
BUN (mg/dl)	17.7 (9.7–24.6)	16.9 (7.1–61.6)	0.457
Cre (mg/dl)	0.76 (0.53–1.25)	0.70 (0.31–8.21)	0.345
T. bil (mg/dl)	1.1 (0.5–3.7)	1.0 (0.3–3.9)	0.563
AST (U/L)	76 (26–340)	63 (23–1971)	0.358
ALT (U/L)	68 (14–339)	56 (13–1574)	0.979
Cholinesterase (U/L)	154 (110–230)	176 (62–301)	0.121
Alb (g/dl)	2.7 (2.0–3.5)	2.6 (1.6–3.8)	0.706
WBC (/µl)	8020 (6040–14,900)	9310 (4940–17,480)	0.649
Hb (g/dl)	11.1 (9.1–13.7)	11.2 (7.4–15.2)	0.937
PLT ( ×10^4^/µl)	17.7 (7.7–36.0)	15.3 (6.5–52.0)	0.501
CRP (mg/dl)	6.70 (5.32–18.24)	5.85 (1.36–17.20)	**0.050**
Max Drain amylase (U/L)^#^	3788 (708–204,240)	2797 (82–41,420)	0.214
Total drain amylase (U)†	555 (78.8–1662)	481 (18.5–16,185)	0.587

CRP: C-reactive protein, NPAG: non-PA formation group PAG: PA formation group

# Max drain amylase was the highest level from the operatively placed abdominal drains

† Total drain amylase was the sum of each drain amylase value * drain output

**Table 5:** Results of blood and drain tests on postoperative day 2

	PAG N = 11	NPAG N = 54	*p-value*
BUN (mg/dl)	13.9 (7.9–29.0)	12.2 (5.6–35.6)	0.170
Cre (mg/dl)	0.87 (0.50–1.26)	0.70 (0.34–5.40)	0.069
T. bil (mg/dl)	1.0 (0.6–4.4)	1.1 (0.3–4.2)	0.661
AST (U/L)	59 (30–256)	56 (20–618)	0.587
ALT (U/L)	51 (13–305)	49 (12–983)	0.909
Cholinesterase (U/L)	124 (90–188)	158 (57–277)	0.059
Alb (g/dl)	2.6 (2.0–3.2)	2.6 (2.0–3.4)	0.410
WBC (/µl)	10,300 (6390–20,870)	11,725 (5710–19,860)	0.740
Hb (g/dl)	11.2 (9.1–14.4)	11.2 (8.0–15.3)	0.944
PLT ( ×10^4^/µl)	17.4 (8.1–38.1)	16.1 (6.8–51.1)	0.637
CRP (mg/dl)	22.17 (12.87–29.54)	14.46 (2.63–28.25)	**0.006**
Max Drain amylase (U/L)^#^	3575 (448–22,900)	3965 (55–41,770)	0.834
Total drain amylase (U)†	428 (51.2–2133)	213 (17.5–2173)	0.306

CRP: C-reactive protein, NPAG: non-PA formation group PAG: PA formation group

# Max drain amylase was the highest level from the operatively placed abdominal drains

† Total drain amylase was the sum of each drain amylase value * drain output

**Table 6:** Results of blood and drain tests on postoperative day 3

	PAG N = 11	NPAG N = 54	*p-value*
BUN (mg/dl)	13.8 (11.7–25.5)	12.9 (6.1–63.6)	0.241
Cre (mg/dl)	0.74 (0.57–1.15)	0.60 (0.33–6.91)	0.076
T. bil (mg/dl)	0.8 (0.5–6.4)	0.9 (0.2–5.0)	0.611
AST (U/L)	37 (18–132)	35 (14–244)	0.937
ALT (U/L)	36 (17–196)	38 (11–464)	0.707
Cholinesterase (U/L)	111 (65–168)	145 (61–270)	**0.031**
Alb (g/dl)	2.4 (1.8–2.8)	2.5 (2.0–3.1)	0.172
WBC (/µl)	9590 (7310–19,500)	10,070 (4860–16,270)	0.958
Hb (g/dl)	10.4 (8.7–15.2)	10.8 (8.5–14.8)	0.720
PLT ( ×10^4^/µl)	18.1 (7.4–42.1)	17.1 (7.5–49.7)	0.766
CRP (mg/dl)	20.93 (15.54–32.68)	14.79 (3.75–32.68)	**0.007**
Max Drain amylase (U/L)^#^	1960 (186–10,320)	1644 (24–17,650)	0.913
Total drain amylase (U)†	80.8 (27.4–304)	76.0 (5.8–1287)	0.832

CRP: C-reactive protein, NPAG: non-PA formation group PAG: PA formation group

# Max drain amylase was the highest level from the operatively placed abdominal drains

† Total drain amylase was the sum of each drain amylase value * drain output

### The cholinesterase-CRP score is a potential predictive marker of PA formation

**Fig. 4:**
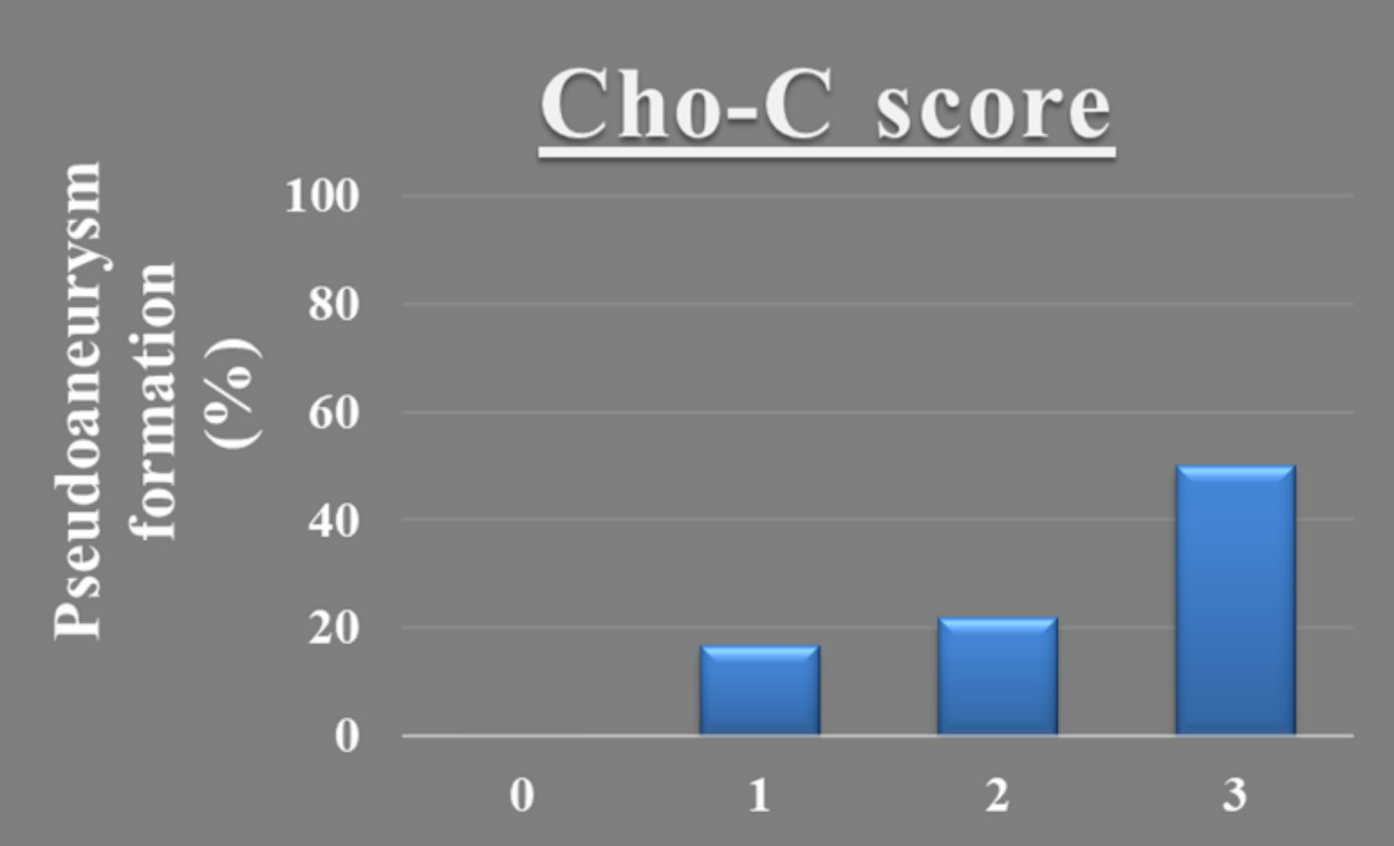
Rate of pseudoaneurysm formation and the Cholinesterase-CRP (Cho-C) score Cho-C score 0: Cholinesterase > 112 U/L and CRP < 16.0 mg/dl on POD 3 Cho-C score 1: Cholinesterase on POD 3 ≤ 112 U/L Cho-C score 2: CRP on POD 3 ≥ 16.0 mg/dl Cho-C score 3: Cholinesterase ≤ 112 U/L and CRP ≥ 16.0 mg/dl on POD 3 Cho-C, Cholinesterase-CRP; POD, postoperative day; CRP, C-reactive protein

We performed multiple logistic regression analysis to identify potential predictive markers of PA formation (Table 7). We found that cholinesterase ≤ 112 U/L and CRP ≥ 16.0 mg/dl on POD 3 were independent predictors of PA formation. We developed the Cholinesterase-CRP (Cho-C) score, obtained from their odds ratios, as follows: score 0: cholinesterase > 112 U/L and CRP < 16.0 mg/dl on POD 3, score 1: cholinesterase on POD 3 ≤ 112 U/L, score 2: CRP on POD 3 ≥ 16.0 mg/dl, score 3: cholinesterase ≤ 112 U/L and CRP ≥ 16.0 mg/dl on POD 3. Importantly, none of the 26 cases with a Cho-C score of 0 developed PA. In contrast, 1 of 6 cases (17%) with Cho-C score 1, and 5 of 23 cases (22%) with Cho-C score 2 had PA formation. Furthermore, PA formation occurred in 5 of 10 cases (50%) with Cho-C score 3 (Fig. 4).

**Table 7:** Multiple logistic regression analysis results of potential predictive markers of PA formation

	Odds ratio	95% CI	*p value*
Age > 70	4.292	0.727–25.64	0.108
Diabetes mellitus	6.066	0.753–48.886	0.090
Cholinesterase on POD 3 ≤ 112 U/L	4.975	1.110–22.22	**0.036**
CRP on POD 3 ≥ 16.0 mg/dl	12.82	1.473–111.1	**0.021**

CI: Confidence interval, CRP: C-reactive protein, PA: pseudoaneurysm, POD: postoperative day

## Discussion

Currently, PD can be performed safely, and the mortality rate is < 5%. However, the severe morbidity rate after PD is > 40% [8]. This study focused on PA formation, which was related to the most life-threatening complication after PD. Next, we developed a novel and simple predictor of PA formation, namely the Cho-C score.

The most common and important postoperative complication after PD is POPF. Clinically relevant POPF (grades B or C) can cause prolonged drain placements, abscesses, hemorrhage, multiorgan dysfunction, and death. Several authors have recently reported that the risk factors for POPF include a soft pancreas, small pancreatic duct, pathology, intra-operative blood loss, male sex, high body mass index (BMI), and surgeon inexperience [6, 19–21]. Various fistula risk scores have been recently proposed for predictive models and have subsequently been widely validated. Since patient-derived risk factors, such as a soft pancreas, small pancreatic duct, and high BMI are unchangeable, POPF is unavoidable, to some extent, in high-risk patients.

PPH is a fatal complication that often requires immediate treatment [9]. However, it occurs in 7.5–10% of patients who undergo surgery, and the mortality rate in those with PPH was reportedly between 16% and 50% [10–12]. Bleeding within 24 h of surgery is considered early bleeding, whereas that after 24 h postoperatively is regarded as late bleeding. Early bleeding is often caused by the technical failure of proper hemostasis during the operation. In contrast, late bleeding is mainly caused by POPF, intra-abdominal abscesses, and bile leaks. Previously, the median onset of late PPH was reported as 10 days (range 6–32 days) in a large-scale cohort study [10].

CRP is a major inflammation marker and can be easily measured. Recently, CRP elevation was reported to be associated with an increased risk of PPH after PD [22]. Therefore, abnormal CRP elevation immediately after PD should be considered uncontrolled inflammation, such as the coexistence of POPF and infection or drainage failure.

Cholinesterase is a short-turnover protein that reflects nutritional status. The half-life of cholinesterase is 11 days, shorter than that of albumin, which is 21 days [23]. Decreased cholinesterase levels reflect malnutrition in patients, which is associated with impaired wound healing [24]. Plasma cholinesterase activity was significantly lower in patients who died than in patients who survived from severe burns [25]. Prealbumin is a rapid-turnover protein with a half-life of 2 days and is commonly used for nutritional assessment [26]. A serum prealbumin level is a sensitive tool for predicting successful engraftment in patients with burns [27]. Although serum prealbumin appears to be more suitable for evaluating wound healing in the early postoperative period, because of the Japanese health insurance system, postoperative prealbumin levels cannot be measured frequently.

Furthermore, optimal nutritional management after PD is under investigation. Enhanced Recovery After Surgery (ERAS) guidelines recommend early oral intake and do not recommend routine use of enteral tube feeding. Meanwhile, a recent meta-analysis reported that percutaneous tube feeding reduced the rate of infectious complications and length of hospital stay. However, these results were derived from all patients with PD rather than from only those with soft pancreas. Recently, we employed a strategy of percutaneous tube feeding according to previously established risk stratification for POPF and delayed gastric emptying [28].

Contrast-enhanced CT is useful to detect vascular abnormalities before PA formation and rupture. Moreover, vascular abnormalities have been reported to be associated with an increased risk of PPH events after PD [22]. Although there are negative results on whether CT should be performed in all patients after PD, it has been pointed out that severe complications could be detected earlier in high-risk patients with POPF [29, 30]. In our experience, the earliest onset of PA rupture and hemorrhagic death was only 5 days postoperatively; therefore, we decided to routinely undertake CT evaluation on PODs 4 or 5. Similarly, the median onset of fatal PPH was previously reported to be around 5 days (132 h) [12]. Based on the results of this study, early CT evaluation is necessary for patients with a positive Cho-C score. Conversely, present study included pseudoaneurysms that occurred more than 1 month postoperatively. Since the CHO-C score is considered an initial postoperative indicator of high inflammation and poor wound healing, repeated CT evaluation should be performed in cases of persistent POPF or intraabdominal infection.

This study had some limitations. First, PD cases were extracted from a single institution over a long period. Although the incidence rates of postoperative morbidity among the surgeons did not differ, the experience of the surgeons varied considerably. Second, the enrolled patients had different physiological and historical backgrounds, and unknown confounding factors and biases could exist. Third, the frequency of POPF and pseudoaneurysms in the present study is higher than the previous report. Finally, this study was retrospective in nature, with a small sample size. Therefore, a prospective study is required to validate these findings.

## Conclusions

Drain amylase levels or total drain amylase content cannot be used to predict PA formation in soft pancreas. The Cho-C score is a simple and useful predictor of PA formation after PD in patients with soft pancreas. In Cho-C score positive cases, regular CT evaluations and strict drain management are necessary to prevent life-threatening hemorrhage.

### Electronic supplementary material

Below is the link to the electronic supplementary material.

Supplementary Material 1

Supplementary Material 2

Supplementary Material 3

## Data Availability

The datasets used and analyzed during the current study are available from the corresponding author on reasonable request.

## Abbreviations

AUC: area under the curve
CRP: C-reactive protein
GNRI: geriatric nutritional risk index
ISGPS: International Study Group on Pancreatic Surgery
mGPS: modified Glasgow prognostic score
PA: pseudoaneurysm
PD: pancreaticoduodenectomy
PNI: prognostic nutritional index
POD: postoperative day
POPF: postoperative pancreatic fistula
PPH: postpancreatectomy hemorrhage
PV: portal vein
ROC: receiver operating characteristic
SMV: superior mesenteric vein
