# Supplementary material for: The cholinesterase and C-reactive protein score is a potential predictor of pseudoaneurysm formation after pancreaticoduodenectomy in patients with soft pancreas
Source: BMC Surg. 2023 Nov 14;23:344. doi: 10.1186/s12893-023-02211-3 (PMC10647161; doi:10.1186/s12893-023-02211-3)
Supplement: Supplementary file 1 — Supplementary Material 1 [file 12893_2023_2211_MOESM1_ESM.docx]

**Supplementary Table 1. Peri-operative clinicopathological characteristics in patients with postoperative pancreatic fistula**

|  | PAG  N = 11 | NPAG  N = 45 | *p-value* |
| --- | --- | --- | --- |
| Age (min-max) (years) | 74 (60–82) | 71 (26–84) | 0.093 |
| Age > 70 (Y : N) | 9 : 2 | 22 : 23 | 0.088 |
| Sex (Male : Female) | 11 : 0 | 24 : 21 | **0.004** |
| Comorbidities (Y : N) | 10 : 1 | 25 : 20 | **0.039** |
| Diabetes mellitus (Y : N) | 5 : 6 | 5 : 40 | **0.018** |
| Cardiovascular disease (Y : N) | 1 : 10 | 19 : 26 | 0.076 |
| Hypertension (Y : N) | 1 : 10 | 17 : 28 | 0.084 |
| ASA score (1 : 2 : 3) | 0 : 9 : 2 | 7 : 32 : 6 | 0.369 |
| Pre-operative BMI (kg/m^2^) (min-max) | 22.4 (19.3–26.8) | 21.6 (16.3–30.8) | 0.813 |
| Primary disease  (Pancreatic cancer : non-pancreatic malignancy^†^ : others) | 3 : 6 : 2 | 13 : 28 : 4 | 0.668 |
| Neoadjuvant chemotherapy (Y : N) | 0 : 11 | 1 : 44 | 1.000 |
| Pre-operative biliary drainage (Y : N) | 6 : 5 | 23 : 22 | 0.838 |
| Pre-operative cholangitis (Y : N) | 3 : 8 | 5 : 40 | 0.181 |
| Operation time (min) (min-max) | 417 (314–652) | 427 (273–754) | 0.523 |
| Blood loss (ml) (min-max) | 705 (194–1520) | 562 (70–3290) | 0.392 |
| Pancreatic duct size (min-max) | 3 (2–4) | 3 (2–12) | 0.775 |
| Fistula risk score (min-max) | 6 (4–9) | 6 (3–9) | 0.584 |
| Bile duct size (mm) (min-max) | 7.5 (4–13) | 8.0 (3–24) | 0.582 |
| Concomitant other organ resection (Y : N) | 0 : 11 | 5 : 40 | 0.571 |
| SMA nerve plexus dissection (Y : N) | 1 : 10 | 11 : 34 | 0.493 |
| Lymph node dissection (D2 : D1 or D0) | 10 : 1 | 41 : 4 | 1.0000 |
| Venous reconstruction (Y : N) | 1 : 10 | 3 : 42 | 1.000 |
| Tube ileostomy (Y : N) | 0 : 11 | 7 : 38 | 0.324 |
| Blood transfusion (Y : N) | 0 : 11 | 3 : 42 | 1.000 |
| Clinically relevant POPF (Y : N) | 11 : 0 | 32 : 13 | 0.051 |
| Chyle leak (Y : N) | 0 : 11 | 0 : 45 | 1.000 |
| Bile leak (Y : N) | 0 : 11 | 1 : 44 | 1.000 |
| Intraabdominal infection (Y : N) | 6 : 5 | 23 : 22 | 0.838 |
| Complication (C.D. grade ≥ Ⅲ) (Y : N) | 11 : 0 | 33 : 12 | 0.097 |
| Mortality (Y : N) | 1 : 10 | 0 : 45 | 0.196 |

ASA: American Society of Anesthesiologists, BMI: body mass index, C.D.: Clavien-Dindo, NPAG: non-PA formation group, PAG: PA formation group, POPF: postoperative pancreatic fistula, SMA: superior mesenteric artery,

^†^ Including 25 biliary tract cancers, 5 neuroendocrine tumors, and 4 duodenal cancers
