# Supplementary material for: The cholinesterase and C-reactive protein score is a potential predictor of pseudoaneurysm formation after pancreaticoduodenectomy in patients with soft pancreas
Source: BMC Surg. 2023 Nov 14;23:344. doi: 10.1186/s12893-023-02211-3 (PMC10647161; doi:10.1186/s12893-023-02211-3)
Supplement: Supplementary file 2 — Supplementary Material 2 [file 12893_2023_2211_MOESM2_ESM.docx]

**Supplementary Table 2. Results of blood and drain tests in patients with postoperative pancreatic fistula on postoperative day 3**

|  | PAG  N = 11 | NPAG  N = 45 | *p-value* |
| --- | --- | --- | --- |
| BUN (mg/dl) | 13.8 (11.7–25.5) | 12.9 (6.1–63.6) | 0.297 |
| Cre (mg/dl) | 0.74 (0.57–1.15) | 0.60 (0.33–6.91) | 0.065 |
| T. bil (mg/dl) | 0.8 (0.5–6.4) | 0.9 (0.3–4.5) | 0.542 |
| AST (U/L) | 37 (18–132) | 36 (14–244) | 0.984 |
| ALT (U/L) | 36 (17–196) | 40 (11–595) | 0.680 |
| Cholinesterase (U/L) | 111 (65–168) | 144 (61–270) | **0.028** |
| Alb (g/dl) | 2.4 (1.8–2.8) | 2.5 (2.0–3.1) | 0.156 |
| WBC (/μl) | 9590 (7310–19500) | 10070 (4860–16270) | 0.893 |
| Hb (g/dl) | 10.4 (8.7–15.2) | 10.8 (8.5–14.8) | 0.710 |
| PLT ( ×10^4^/μl) | 18.1 (7.4–42.1) | 17.6 (9.0–29.7) | 0.869 |
| CRP (mg/dl) | 20.93 (15.54–32.68) | 14.79 (4.45–32.68) | **0.009** |
| Max Drain amylase (U/L)^#^ | 1960 (186–10320) | 2529 (307–17650) | 0.388 |
| Total drain amylase (U)† | 80.8 (27.4–304) | 87.6 (11.7–1287) | 0.768 |

CRP: C-reactive protein, NPAG: non-PA formation group PAG: PA formation group

# Max drain amylase was the highest level from the operatively placed abdominal drains.

† Total drain amylase was the sum of each drain amylase value * drain output.
