# Supplementary material for: The cholinesterase and C-reactive protein score is a potential predictor of pseudoaneurysm formation after pancreaticoduodenectomy in patients with soft pancreas
Source: BMC Surg. 2023 Nov 14;23:344. doi: 10.1186/s12893-023-02211-3 (PMC10647161; doi:10.1186/s12893-023-02211-3)
Supplement: Supplementary file 3 — Supplementary Material 3 [file 12893_2023_2211_MOESM3_ESM.docx]

**Supplementary Table 3. Multiple logistic regression analysis results of potential predictive markers of PA formation in patients with postoperative pancreatic fistula**

|  | **Odds ratio** | **95% CI** | ***p value*** |
| --- | --- | --- | --- |
| Age >70 | 8.333 | 0.769–90.91 | 0.081 |
| Diabetes mellitus | 6.757 | 0.609–76.923 | 0.120 |
| Cholinesterase on POD 3 ≤112 U/L | 7.092 | 1.361–37.04 | **0.020** |
| CRP on POD 3 ≥16.0 mg/dl | 16.67 | 1.739–166.7 | **0.015** |

CI: Confidence interval, CRP: C-reactive protein, PA: pseudoaneurysm, POD: postoperative day
